# Rokumi-jio-gan-Containing Prescriptions Attenuate Oxidative Stress, Inflammation, and Apoptosis in the Remnant Kidney

**DOI:** 10.1155/2012/587902

**Published:** 2012-11-29

**Authors:** Chan Hum Park, Sul Lim Lee, Takuya Okamoto, Takashi Tanaka, Takako Yokozawa

**Affiliations:** ^1^Institute of Natural Medicine, University of Toyama, Toyama 930-0194, Japan; ^2^Chinese Medicine and Health Food Department, Iskra Industry Co., Ltd., Tokyo 103-0027, Japan; ^3^Graduate School of Biomedical Sciences, Nagasaki University, Nagasaki 852-8521, Japan; ^4^Organization for Promotion of Regional Collaboration, University of Toyama, Toyama 930-8555, Japan

## Abstract

Two Rokumi-jio-gan-containing prescriptions (Hachimi-jio-gan and Bakumi-jio-gan) were selected to examine their actions in nephrectomized rats. Each prescription was given orally to rats for 10 weeks after the excision of five-sixths of their kidney volumes, and its effect was compared with non-nephrectomized and normal rats. Rats given Hachimi-jio-gan and Bakumi-jio-gan showed an improvement of renal functional parameters such as serum urea nitrogen, creatinine, creatinine clearance, and urinary protein. The nephrectomized rats exhibited the up-regulation of nicotinamide adenine dinucleotide phosphate oxidase subunits, c-Jun N-terminal kinase (JNK), phosphor-JNK, c-Jun, transforming growth factor-**β**_1_, nuclear factor-kappa B, cyclooxygenase-2, inducible nitric oxide synthase, monocyte chemotactic protein-1, intracellular adhesion molecule-1, Bax, cytochrome *c*, and caspase-3, and down-regulation of NF-E2-related factor 2, heme oxygenase-1, and survivin; however, Bakumi-jio-gan administration acts as a regulator in inflammatory reactions caused by oxidative stress in renal failure. Moreover, the JNK pathway and apoptosis-related protein expressions, Bax, caspase-3, and survivin, were ameliorated to the normal levels by Hachimi-jio-gan administration. The development of renal lesions, glomerular sclerosis, tubulointerstitial damage, and arteriolar sclerotic lesions, estimated by histopathological evaluation and scoring, was strong in the groups administered Hachimi-jio-gan rather than Bakumi-jio-gan. This study suggests that Rokumi-jio-gan-containing prescriptions play a protective role in the progression of renal failure.

## 1. Introduction

Chronic kidney disease is a worldwide public health problem. In Japan, there is a rising incidence and prevalence of kidney failure, with poor outcomes and a high medical cost. The incidence and prevalence of end-stage renal disease have doubled in the past 10 years and are expected to continue to rise steadily in the future.

 The prognosis of patients with chronic renal failure has markedly improved along with the establishment of hemodialysis therapy and advances in medical care technology. However, the continuation of maintenance dialysis is a marked burden on patients from both mental and physical aspects, and social problems including financial issues have arisen due to the increase in the number of dialysis patients. Under these circumstances, various conservative therapies are available for chronic renal failure, such as a low-protein, high-caloric diet, essential amino acid therapy, and the administration of activated charcoal or lactulose [1–3]. Although these treatment methods show some clinical effects, methods other than dialysis and renal transplantation are still urgently needed for the treatment of chronic renal failure.

 Traditional medicines, including Chinese prescriptions, have attracted much attention due to their extensive and unique biological activities without toxicity and/or side effects [4]. Hachimi-jio-gan has been long and widely used to treat several chronic diseases, including nephritis, infertility diabetes, and vegetative ataxia [5], although scientific evidence supporting the pharmacological basis for its therapeutic effects has not yet been well established. In particular, it has been widely used to treat renal dysfunction in human subjects with glomerulonephritis, hypertension, and nephritic syndrome [6]. Our previous studies demonstrated that Hachimi-jio-gan alleviated the pathological conditions of diabetic nephropathy in an experimental model [7–10]. However, the effect of Hachimi-jio-gan on chronic renal failure-induced alterations such as oxidative stress, apoptosis, inflammation, and/or morphological changes in the kidney of nephrectomized rats has not yet been studied.

 Hachimi-jio-gan is composed of Rokumi-jio-gan, Cinnamomi Cortex, and Aconiti Tuber. On the other hand, Bakumi-jio-gan is also composed of Rokumi-jio-gan contained in Hachimi-jio-gan. These two prescriptions are expected to provide a novel therapeutic approach to nephrectomy. Therefore, we employed an animal model of nephrectomy, in which rats were subjected to subtotal nephrectomy, and administered Hachimi-jio-gan and Bakumi-jio-gan for 10 weeks to investigate its effect on the oxidative stress, inflammation, and apoptosis associated with nephrectomy.

## 2. Materials and Methods

### 2.1. Materials

Protease inhibitor mixture solution, ethylenediaminetetraacetic acid (EDTA), and 10% neutral-buffered formalin were purchased from Wako Pure Chemical Industries, Ltd. (Osaka, Japan). 2′,7′-Dichlorofluorescein diacetate (DCFH-DA) was purchased from Molecular Probes (Eugene, OR, USA). The Bio-Rad protein assay kit and pure nitrocellulose membrane were purchased from Bio-Rad Laboratories (Tokyo, Japan). *β*-Actin and phenylmethylsulfonyl fluoride (PMSF) were purchased from Sigma Chemical Co. (St. Louis, MO, USA). Rabbit polyclonal antibodies against p22^phox^, NF-E2-related factor 2 (Nrf2), heme oxygenase-1 (HO-1), transforming growth factor-*β*_1_ (TGF-*β*_1_), nuclear factor-kappa B (NF-*κ*B)p65, monocyte chemotactic protein-1 (MCP-1), intracellular adhesion molecule-1 (ICAM-1), Bax, and cytochrome *c*, and mouse monoclonal antibody against cyclooxygenase-2 (COX-2), inducible nitric oxide synthase (iNOS), and histone were purchased from Santa Cruz Biotechnology, Inc. (Santa Cruz, CA, USA). Rabbit polyclonal antibodies against c-Jun N-terminal kinase (JNK) and phosphor (p)-JNK, and mouse monoclonal antibody against AP-1 (c-Jun) were purchased from Cell Signaling Technology (Danvers, MA, USA). Poly anti-NADPH oxidase-4 (Nox-4) (LifeSpan BioSciences, Seattle, WA, USA), mono anti-caspase-3 (BioVision, Mountain View, CA, USA), and survivin (Novus Biologicals, Littleton, CO, USA) were also used. Goat anti-rabbit and goat anti-mouse IgG horseradish peroxidase (HRP)-conjugated secondary antibodies were purchased from Santa Cruz Biotechnology, Inc. ECL Western Blotting Detection Reagents were purchased from GE Healthcare (Piscataway, NJ, USA).

### 2.2. Hachimi-jio-gan and Bakumi-jio-gan Extracts

**Figure 1:**
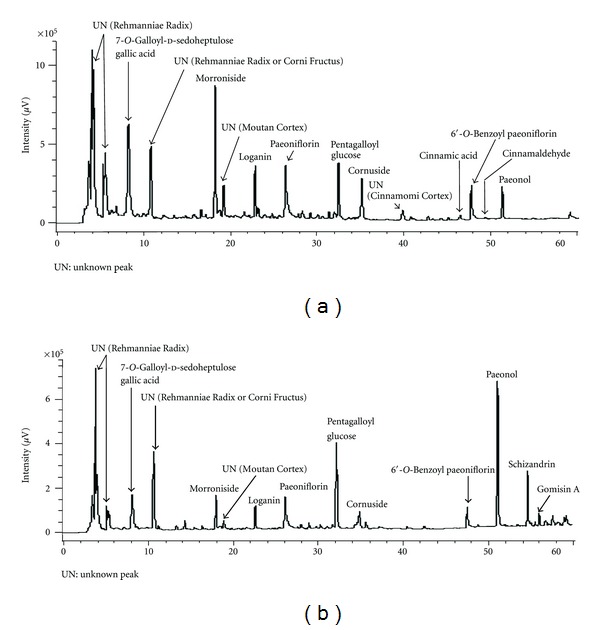
HPLC profile of Hachimi-jio-gan (a) and Bakumi-jio-gan (b) extracts.

The composition of Hachimi-jio-gan used in this study was as follows (figures indicate proportions of each ingredient, expressed in parts per whole): Rehmanniae Radix (*Rehmannia glutinosa* Li bosch. var. *purpurea* Makino) 6, Corni Fructus (*Cornus officinalis* Sieb. et Zucc.) 3, Dioscoreae Rhizoma (*Dioscorea japonica* Thunb.) 3, Alismatis Rhizoma (*Alisma orientale* Juzep.) 3, Hoelen (*Poria cocos* Wolf) 3, Moutan Cortex (*Paeonia suffruticosa* Andrews) 2.5, Cinnamomi Cortex (*Cinnamomum cassia* Blume) 1, Aconiti Tuber (*Aconitum carmichaeli* Debx) 0.5. These eight crude drugs were boiled gently in 10 times their volume of water for 60 min, filtered, and the filtrate was spray-dried to obtain the extract at a yield of about 10%, by weight, of the original preparation. The composition of Bakumi-jio-gan was: Rehmanniae Radix (*Rehmannia glutinosa* Libosch. var. *purpurea* Makino) 0.75, Corni Fructus (*Cornus officinalis* Sieb. et Zucc.) 0.37, Dioscoreae Rhizoma (*Dioscorea japonica* Thunb.) 0.37, Alismatis Rhizoma (*Alisma orientale* Juzep.) 0.28, Hoelen (*Poria cocos* Wolf) 0.28 g; Moutan Cortex (*Paeonia suffruticosa* Andrews) 0.28, Schisandrae Fructus (*Schisandra chinensis* Baillon) 0.199, Ophiopogonis Tuber (*Ophiopogon japonicus* Ker-Gawler var. *genuinus* Maxim) 0.28. As Hachimi-jio-gan described above, the extract was prepared at a yield of about 12%, by weight, of the original crude drugs. For analysis of the components, Hachimi-jio-gan and Bakumi-jio-gan were separately pulverized and a portion (0.5 g) was extracted with 5 mL of methanol at room temperature for 8 h and then sonicated for 20 min. After filtration with a membrane filter (0.45 *μ*m), an aliquot (10 *μ*L) was analyzed by high-performance liquid chromatography (HPLC). HPLC was performed on a Cosmosil 5C_18_-AR II (Nacalai Tesque, Kyoto, Japan) column (250 × 4.6 mm i.d) with gradient elution from 4 to 30% (39 min) and from 30 to 75% (15 min) CH_3_CN in 50 mM H_3_PO_4_ at 40°C. The flow rate was 0.8 mL/min and the effluent from the column was monitored and processed in the JASCO photodiode array detector MD-2010. All assigned peaks were identified by comparing their UV spectral data with those co-injected authentic samples using Class LC-10 Version 1.62 software (Shimadzu, Kyoto, Japan). The HPLC profile of Hachimi-jio-gan extract is shown in Figure 1(a). Morroniside, gallic acid, loganin, paeoniflorin, and pentagalloyl glucose were the major components of Hachimi-jio-gan; corunuside, cinnamic acid, 6′-*O*-benzoyl paeoniflorin, cinnamaldehyde, and paeonol were also detected. In Bakumi-jio-gan extract, paeonol, pentagalloyl glucose, schizandrin, gallic acid, morroniside, paeoniflorin, loganin, 6′-*O*-benzoyl paeoniflorin, cornuside, and gomisin A were detected (Figure 1(b)).

### 2.3. Animals and Treatment

Male Wistar rats (Shizuoka Agricultural Cooperative Association for Laboratory Animals, Hamamatsu, Japan), each weighing about 200 g, underwent resection of 2/3 of the left kidney and total excision of the right kidney with an interoperative interval of 12–14 days. The blood urea nitrogen level of the rats was determined after recovery from the operation, and they were divided into three groups so that any intergroup difference in the blood urea nitrogen level was avoided. The first group was given water, while the other two groups were given Hachimi-jio-gan 150 mg/kg body weight/day or Bakumi-jio-gan 150 mg/kg body weight/day orally for 10 consecutive weeks. To ensure that the food consumption was as constant as possible among the three groups, they were raised on a commercial chow (CLEA Japan Inc., Tokyo, Japan; type CE-2) using a pair-feeding schedule.

### 2.4. Determination of Blood and Urine Component Levels

Blood urea nitrogen and creatinine (Cr) were determined using commercial reagents (BUN Kainos and CRE-EN Kainos obtained from Kainos Laboratories, Inc., Tokyo, Japan). Urine component levels were determined as follows: Cr using a commercial reagent (CRE-EN Kainos) and protein by the sulfosalicylic acid method [11]. Creatinine clearance (*C*_Cr_) was calculated on the basis of the urinary Cr, serum Cr, urine volume, and body weight values using the following equation: *C*_Cr_ (mL/min/kg body weight) = [urinary Cr (mg/dL) × urine volume (mL)/serum Cr (mg/dL)] × [1,000/body weight (g)] × [1/1,440 (min)]. The serum reactive oxygen species (ROS) generation level was determined using the method of Ali et al. [12].

### 2.5. Assessment of Renal ROS Generation

ROS generation was measured employing the method of Ali et al. [12]. Renal tissues were homogenized on ice with 1 mM EDTA-50 mM sodium phosphate buffer (pH 7.4), and then 25 mM DCFH-DA was added to homogenates. After incubation for 30 min, the changes in fluorescence values were determined at an excitation wavelength of 486 nm and emission wavelength of 530 nm.

### 2.6. Preparation of Nuclear and Post-Nuclear Fractions

Nuclear protein extraction was performed according to the method of Komatsu [13]. In brief, renal tissues were homogenized with ice-cold lysis buffer containing 5 mM Tris-HCl (pH 7.5), 2 mM MgCl_2_, 15 mM CaCl_2_, and 1.5 M sucrose, and then 0.1 M dithiothreitol (DTT) and protease inhibitor mixture solution were added. After centrifugation (10,500 ×g for 20 min at 4°C), the pellet was suspended with extraction buffer containing 20 mM 2-[4-(2-hydroxyethyl)-1-piperazyl] ethanesulfonic acid (pH 7.9), 1.5 mM MgCl_2_, 0.42 M NaCl, 0.2 mM EDTA, and 25% (v/v) glycerol, and then 0.1 M DTT and protease inhibitor mixture solution were added. The mixture was placed on ice for 30 min. The nuclear fraction was prepared by centrifugation at 20,500 ×g for 5 min at 4°C.

 The postnuclear fraction was extracted from the kidney of each mouse as described below. In brief, renal tissue was homogenized with ice-cold lysis buffer (pH 7.4) containing 137 mM NaCl, 20 mM Tris-HCl, 1% Tween 20, 10% glycerol, 1 mM PMSF, and protease inhibitor mixture solution. The homogenate was then centrifuged at 2,000 ×g for 10 min at 4°C. The protein concentration in each fraction was determined using a Bio-Rad protein kit (Bio-Rad Laboratories, Hercules, CA, USA).

### 2.7. Immunoblotting Analyses

 For the determination of Nrf2, c-Jun, NF-*κ*Bp65, and histone, 15 *μ*g of protein from each nuclear fraction was electrophoresed through an 8% sodium dodecylsulfate polyacrylamide gel (SDS-PAGE). Separated proteins were transferred to a nitrocellulose membrane, blocked with 5% (w/v) skim milk solution for 1 h, and then incubated with primary antibodies to Nrf2, c-Jun, NF-*κ*Bp65, and histone, respectively, overnight at 4°C. After the blots were washed, they were incubated with anti-rabbit or anti-mouse IgG HRP-conjugated secondary antibody for 1.5 h at room temperature. Also, 10 *μ*g of protein of each post-nuclear fraction of Nox-4, p22^phox^, HO-1, JNK, p-JNK, TGF-*β*_1_, COX-2, iNOS, MCP-1, ICAM-1, Bax, cytochrome *c*, caspase-3, survivin, and *β*-actin was electrophoresed through 8–15% SDS-PAGE. Each antigen-antibody complex was visualized using ECL Western Blotting Detection Reagents and detected by chemiluminescence with LAS-4000 (Fujifilm, Tokyo, Japan). Band densities were determined using ATTO Densitograph Software (ATTO Corporation, Tokyo, Japan) and quantified as the ratio to histone or *β*-actin. The protein levels of groups are expressed relative to those of normal rats (represented as 1).

### 2.8. Histopathological Evaluation and Morphological Changes

Renal tissues were fixed in 10% neutral formalin solution, embedded in paraffin, and cut into semithin sections (2 *μ*m thick), which were stained with hematoxylin and eosin (HE), periodic acid-Schiff's reagent (PAS), periodic acid-methenamine silver (PAM), and phosphotungstic acid-hematoxylin (PTAH). Two hundred or fewer glomeruli from each sample were examined by light microscopy. The score of global glomerulosclerosis was as follows: no global glomerulosclerosis = 0, <10% = 1, 10–30% = 2, 30–50% = 3, and >50% = 4. The degree of tubulointerstitial changes was classified by the extent of the fibrotic area, and tubular atrophy and/or widening and graded 0 to 4. Mesangial matrix expansion was graded from 0 to 4 according to the degree of increased amounts of PAS-positive material in the mesangial region compared with normal samples. The interlobular and efferent arteriolar sclerotic changes (thickness of media and stenosis of lumen) were graded from 0 to 4. Evaluation of histological findings was conducted without knowledge of the source of the samples.

### 2.9. Statistical Analysis

Data are expressed as mean ± S.E.M. Significance was assessed by one-way analysis of variance (ANOVA) followed by Dunnett's multiple comparison test (SPSS 11.5.1 for Windows, 2002, SPSS Inc., USA). Values of *P* < 0.05 were considered significant.

## 3. Results

### 3.1. Body Weight Changes, Food and Water Consumption

**Figure 2:**
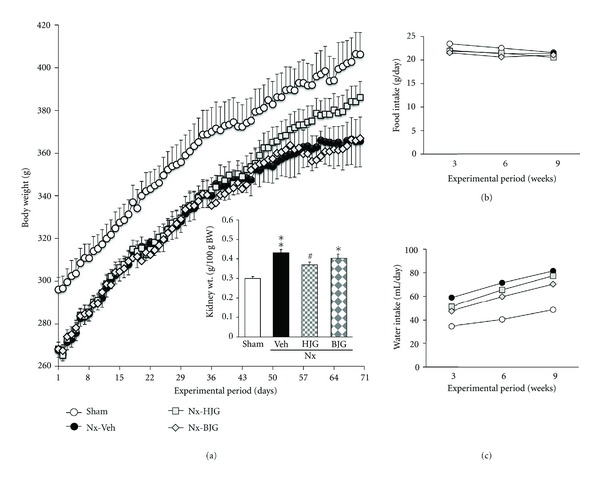
Body weight changes, food and water consumption. Sham, sham rats; Nx-Veh, nephrectomized vehicle-treated rats; Nx-HJG, nephrectomized Hachimi-jio-gan-treated rats; Nx-BJG, nephrectomized Bakumi-jio-gan-treated rats. Data are the mean ± S.E.M. Significance: *P < 0.05, **P < 0.001 versus sham rats; ^#^ *P* < 0.05 versus nephrectomized vehicle-treated rats.

Figure 2 shows the changes in the body weight, food and water intakes, and kidney weight during the experimental period. The nephrectomized control rats displayed a marked decrease in body weight, and the decreased body weight was slightly increased, not significantly, by the Hachimi-jio-gan administration. At the end of the study, the kidney weight in nephrectomized control rats was 1.4 times greater than that in sham-treated rats, but was significantly decreased by Hachimi-jio-gan administration. However, the food and water intakes were not changed by Hachimi-jio-gan treatment. Also, compared with nephrectomized control rats, the body weight and food and water intakes were not changed by Bakumi-jio-gan treatment.

### 3.2. Renal Functional Parameters

**Figure 3:**
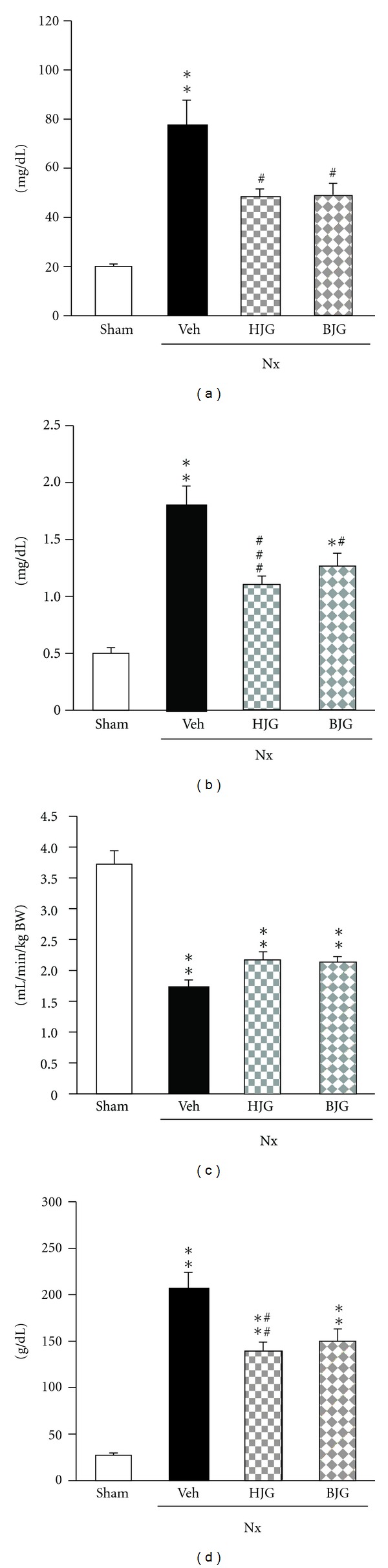
Renal functional parameters. (a) Blood urea nitrogen, (b) serum Cr, (c) *C*_Cr_, and (d) urinary protein. Sham, sham rats; Nx-Veh, nephrectomized vehicle-treated rats; Nx-HJG, nephrectomized Hachimi-jio-gan-treated rats; Nx-BJG, nephrectomized Bakumi-jio-gan-treated rats. Data are the mean ± S.E.M. Significance: *P < 0.01, **P < 0.001 versus sham rats; ^#^ *P* < 0.05, ^##^ *P* < 0.01, ^###^ *P* < 0.001 versus nephrectomized vehicle-treated rats.

Figure 3 represents the effect of Hachimi-jio-gan and Bakumi-jio-gan on the renal functional parameters of serum and urine by nephrectomy. In the nephrectomized control rats, the blood urea nitrogen level was significantly increased to reach 77.5 mg/dL at 10 weeks (18.5 mg/dL in normal rats), while it was reduced significantly to 47.0 mg/dL by the administration of 150 mg/kg body weight/day Hachimi-jio-gan. The oral administration of Bakumi-jio-gan for 10 weeks also caused a 22% decrease in the level of urea nitrogen as compared with that in the control rats (Figure 3(a)). Moreover, the higher level of serum Cr in nephrectomized control rats compared with normal rats decreased with the administration of Hachimi-jio-gan and Bakumi-jio-gan (Figure 3(b)). As shown in Figure 3(c), *C*_Cr_ in nephrectomized control rats was significantly lower than in normal rats, from 3.69 to 1.74 mL/min/kg body weight, while the administration of Hachimi-jio-gan and Bakumi-jio-gan at 150 mg/kg body weight/day elevated the value to 2.23 and 2.20 mL/min/kg body weight, respectively. The urinary protein excretion of nephrectomized control rats was 8.5-fold higher than that of normal rats. The elevation in urinary protein in nephrectomized control rats was reduced with the administration of Hachimi-jio-gan and Bakumi-jio-gan (Figure 3(d)).

### 3.3. Biomarkers Associated with Oxidative Stress in the Serum and Kidney

**Figure 4:**
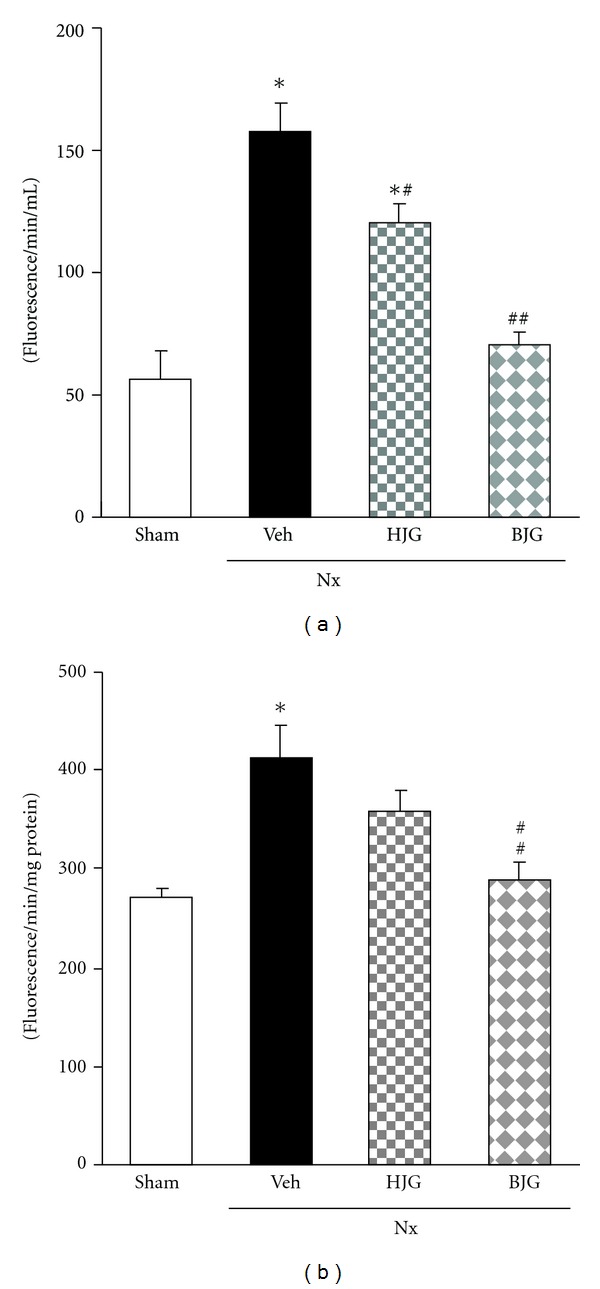
ROS generation levels. (a) Serum and (b) renal tissues. Sham, sham rats; Nx-Veh, nephrectomized vehicle-treated rats; Nx-HJG, nephrectomized Hachimi-jio-gan-treated rats; Nx-BJG, nephrectomized Bakumi-jio-gan-treated rats. Data are the mean ± S.E.M. Significance: *P < 0.001 versus sham rats; ^#^ *P* < 0.05, ^##^ *P* < 0.001 versus nephrectomized vehicle-treated rats.

As shown in Figure 4(a), the serum level of ROS in nephrectomized control rats was significantly higher than that in normal rats, whereas, by the administration of Bakumi-jio-gan, the elevated level was markedly decreased nearly to the level of normal rats. The oral administration of Hachimi-jio-gan also led to a significant decrease. In addition, the Bakumi-jio-gan-treated rats showed a significant decrease in renal ROS; however, there were no differences between nephrectomized control and Hachimi-jio-gan-treated groups (Figure 4(b)).

### 3.4. Nox-4 and p22^phox^ Protein Expressions in Renal Tissues

**Figure 5:**
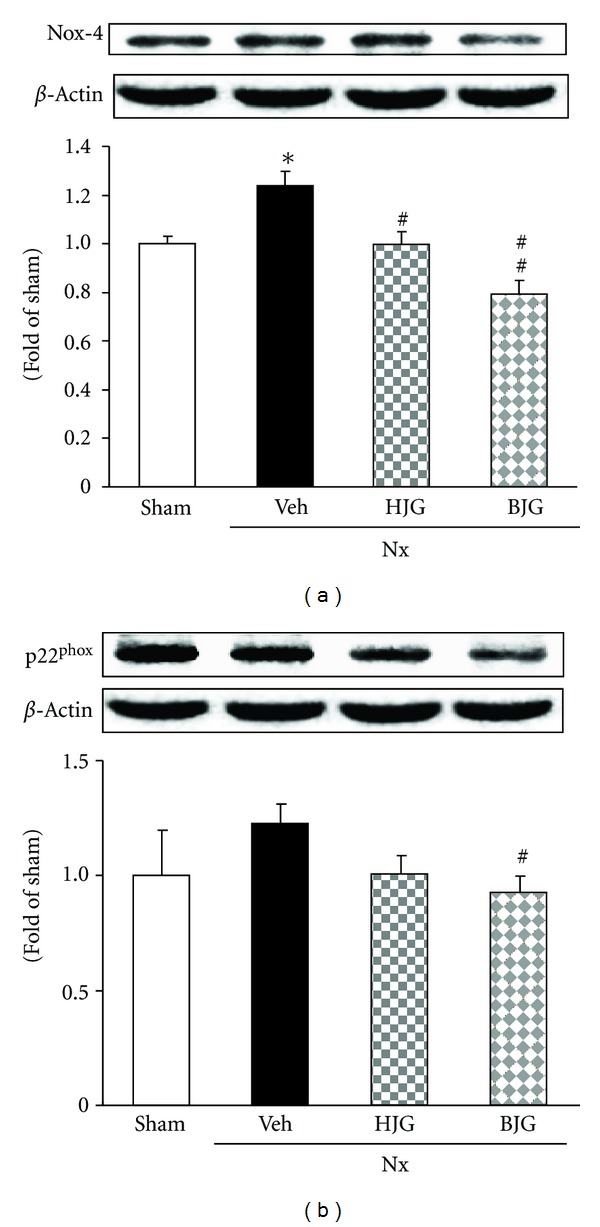
Renal oxidative stress-related protein expressions. Representative immunoblots for (a) Nox-4 and (b) p22^phox^. Immunoblotting analyses were performed as described in Materials and Methods. Sham, sham rats; Nx-Veh, nephrectomized vehicle-treated rats; Nx-HJG, nephrectomized Hachimi-jio-gan-treated rats; Nx-BJG, nephrectomized Bakumi-jio-gan-treated rats. Data are the mean ± S.E.M. Significance: *P < 0.001 versus sham rats; ^#^ *P* < 0.05, ^##^ *P* < 0.01 versus nephrectomized vehicle-treated rats.

The effects of Hachimi-jio-gan and Bakumi-jio-gan on renal Nox-4 and p22^phox^ protein expressions in nephrectomized rats are shown in Figure 5. Nox-4 expression in the kidney of nephrectomized rats was significantly increased compared with that in sham-treated rats (*P* < 0.001). However, 150 mg/kg Bakumi-jio-gan treatment was decreased below the normal level. The administration of Hachimi-jio-gan was also significantly decreased; however, the strong downregulation of Nox-4 protein was observed in the group administered Bakumi-jio-gan, as shown in Figure 5(a). Regarding p22^phox^ protein expression, there was no alteration between the sham and nephrectomized control rats; however, Bakumi-jio-gan administration showed a significant decrease. Hachimi-jio-gan showed a tendency to decrease the p22^phox^ protein expression (without significance), compared with nephrectomized control rats (Figure 5(b)).

### 3.5. Nrf2 and HO-1 Protein Expressions in Renal Tissues

**Figure 6:**
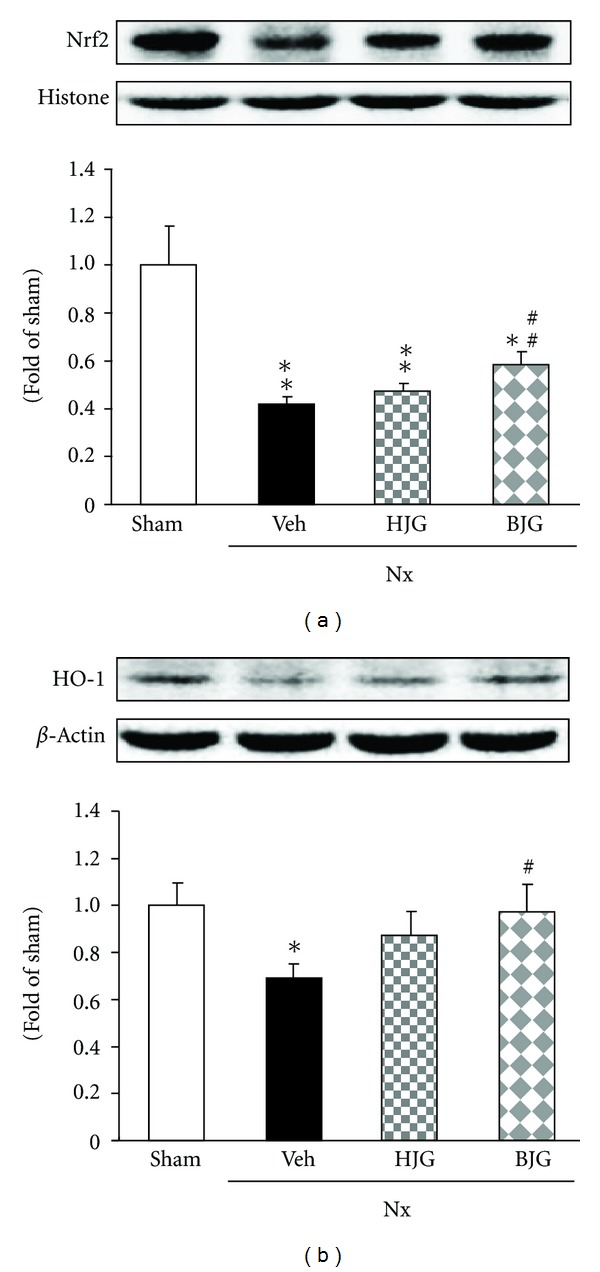
Renal oxidative stress-related protein expressions. Representative immunoblots for (a) Nrf2 and (b) HO-1. Immunoblotting analyses were performed as described in Materials and Methods. Sham, sham rats; Nx-Veh, nephrectomized vehicle-treated rats; Nx-HJG, nephrectomized Hachimi-jio-gan-treated rats; Nx-BJG, nephrectomized Bakumi-jio-gan-treated rats. Data are the mean ± S.E.M. Significance: *P < 0.05, **P < 0.001 versus sham rats; *P < 0.05, **P < 0.01 versus nephrectomized vehicle-treated rats.

As shown in Figure 6, Nrf2 and HO-1 expressions in the kidney of nephrectomized control rats were significantly decreased compared with those in sham rats. These decreased protein expressions were significantly up-regulated by Bakumi-jio-gan treatment. Nrf2 and HO-1 protein expressions in Hachimi-jio-gan-treated rats were slightly increased, without significance (Figures 6(a), 6(b)).

### 3.6. JNK, p-JNK, c-Jun, and TGF-*β*_1_ Protein Expressions in Renal Tissues

**Figure 7:**
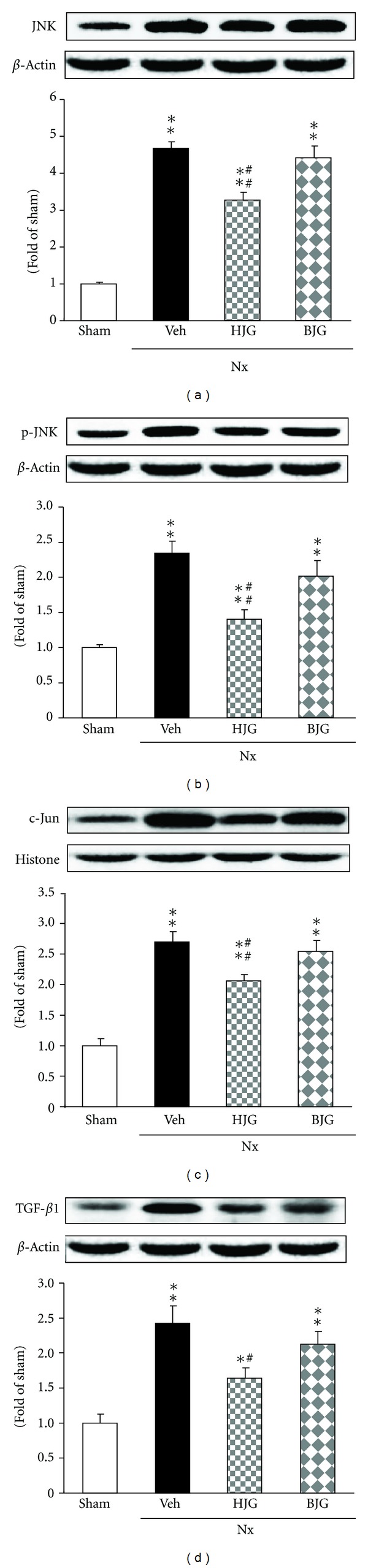
Renal oxidative stress- and inflammation-related protein expressions. Representative immunoblots for (a) JNK, (b) p-JNK, (c) c-Jun, and (d) TGF-*β*_1_. Immunoblotting analyses were performed as described in Materials and Methods. Sham, sham rats; Nx-Veh, nephrectomized vehicle-treated rats; Nx-HJG, nephrectomized Hachimi-jio-gan-treated rats; Nx-BJG, nephrectomized Bakumi-jio-gan-treated rats. Data are the mean ± S.E.M. Significance: *P < 0.05, **P < 0.001 versus sham rats; ^#^ *P* < 0.05, ^##^ *P* < 0.01 versus nephrectomized vehicle-treated rats.

The protein expressions of JNK, p-JNK, c-Jun, and TGF-*β*_1_ in nephrectomized control rats were significantly induced to a greater extent compared with sham rats (Figure 7). The administration of Hachimi-jio-gan led to a significant down-regulation of these four protein expressions. The elevated levels in nephrectomized control rats were also reduced with the administration of Bakumi-jio-gan; however, a stronger down-regulation of these four proteins was observed in the group administered Hachimi-jio-gan rather than Bakumi-jio-gan.

### 3.7. NF-*κ*Bp65, COX-2, and iNOS Protein Expressions in Renal Tissues

**Figure 8:**
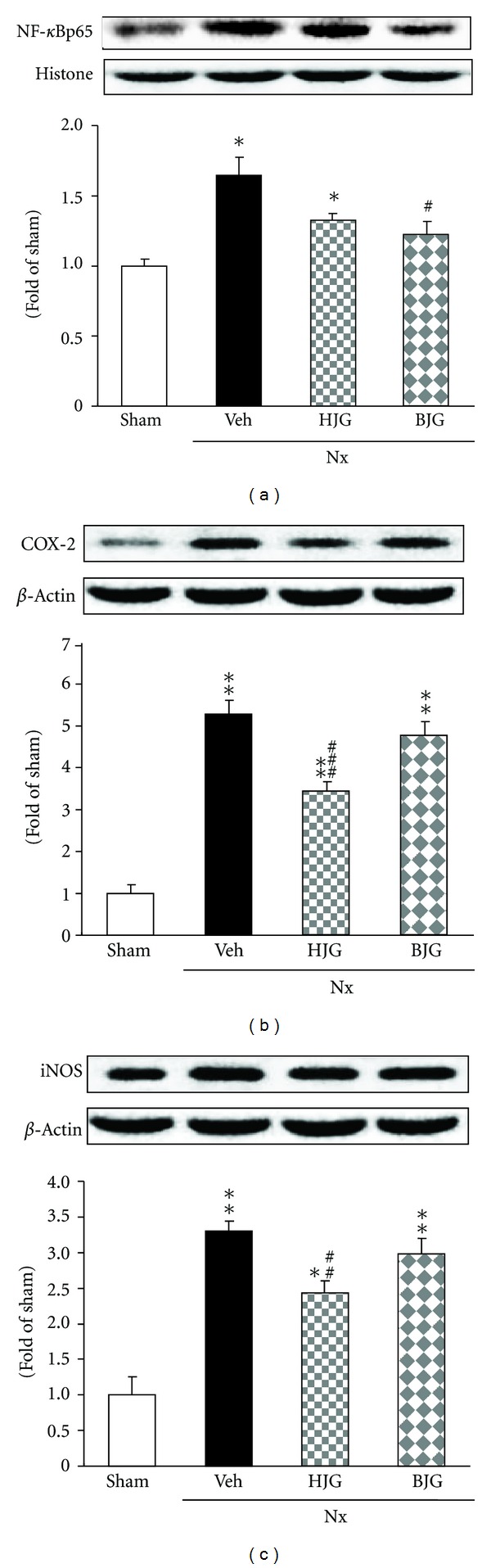
Renal inflammation-related protein expressions. Representative immunoblots for (a) NF-*κ*Bp65, (b) COX-2, and (c) iNOS. Immunoblotting analyses were performed as described in Materials and Methods. Sham, sham rats; Nx-Veh, nephrectomized vehicle-treated rats; Nx-HJG, nephrectomized Hachimi-jio-gan-treated rats; Nx-BJG, nephrectomized Bakumi-jio-gan-treated rats. Data are the mean ± S.E.M. Significance: *P < 0.01, **P < 0.001 versus sham rats; ^#^ *P* < 0.05, ^##^ *P* < 0.01, ^###^ *P* < 0.001 versus nephrectomized vehicle-treated rats.

The nephrectomized control rats showed the up-regulation of nuclear NF-*κ*Bp65 protein and also COX-2 and iNOS proteins compared with sham rats (Figure 8). In the Bakumi-jio-gan-treated group, NF-*κ*Bp65 protein expression was significantly decreased (Figure 8(a)), and the Hachimi-jio-gan-treated group showed a significant change in COX-2 and iNOS (Figures 8(b), 8(c)).

### 3.8. MCP-1 and ICAM-1 Protein Expressions in Renal Tissues

**Figure 9:**
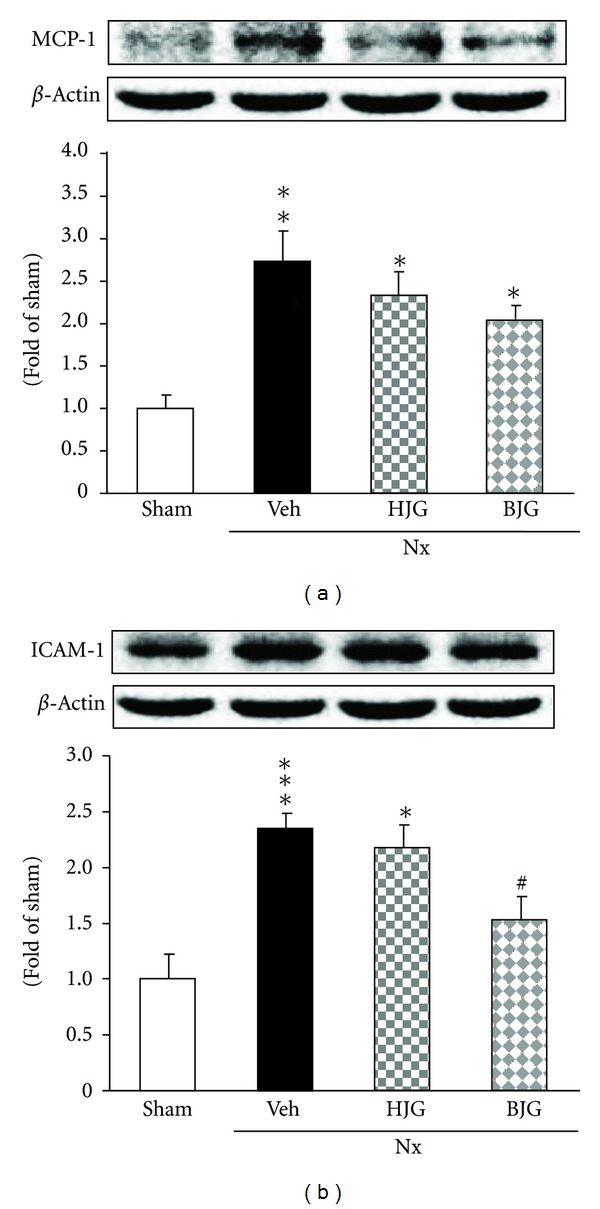
Renal inflammation-related protein expressions. Representative immunoblots for (a) MCP-1 and (b) ICAM-1. Immunoblotting analyses were performed as described in Materials and Methods. Sham, sham rats; Nx-Veh, nephrectomized vehicle-treated rats; Nx-HJG, nephrectomized Hachimi-jio-gan-treated rats; Nx-BJG, nephrectomized Bakumi-jio-gan-treated rats. Data are the mean ± S.E.M. Significance: *P < 0.05, **P < 0.01, ***P < 0.001 versus sham rats; ^#^ *P* < 0.05 versus nephrectomized vehicle-treated rats.

Renal MCP-1 and ICAM-1 protein expressions were examined. As shown in Figure 9, MCP-1 and ICAM-1 expressions in nephrectomized control rats were significantly increased compared to those in sham rats, but MCP-1 expression of Hachimi-jio-gan- and Bakumi-jio-gan-treated nephrectomized rats was slightly decreased, and ICAM-1 expression in Bakumi-jio-gan-treated rats exhibited a significant decrease from the value of nephrectomized control rats. In contrast, Hachimi-jio-gan treatment led to no changes in ICAM-1 protein expression.

### 3.9. Bax, Cytochrome *c*, Caspase-3, and Survivin Protein Expressions in Renal Tissues

**Figure 10:**
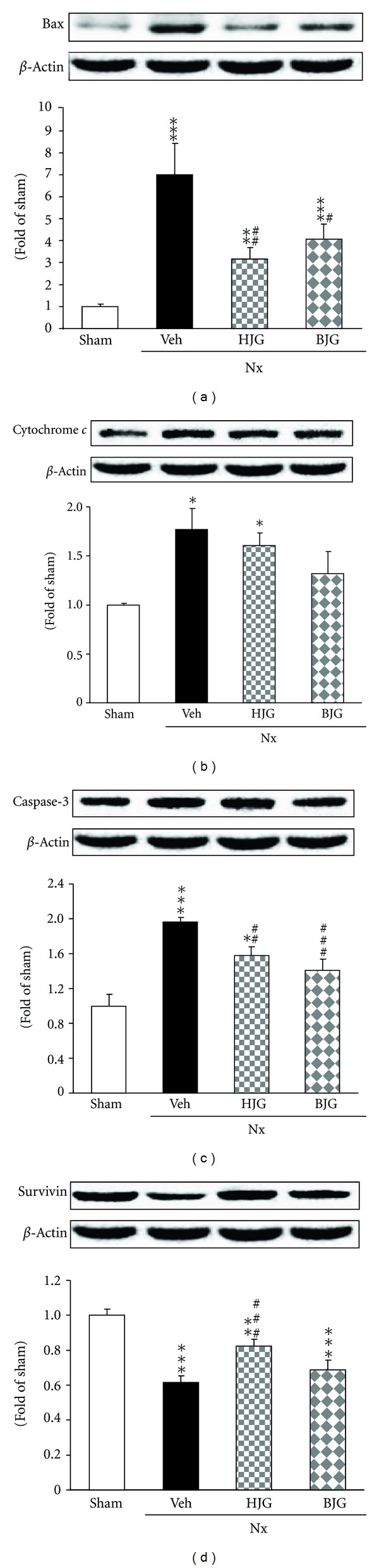
Renal apoptosis-related protein expressions. Representative immunoblots for (a) Bax, (b) cytochrome *c*, (c) caspase-3, and (d) survivin. Immunoblotting analyses were performed as described in Materials and Methods. Sham, sham rats; Nx-Veh, nephrectomized vehicle-treated rats; Nx-HJG, nephrectomized Hachimi-jio-gan-treated rats; Nx-BJG, nephrectomized Bakumi-jio-gan-treated rats. Data are the mean ± S.E.M. Significance: *P < 0.05, **P < 0.01, ***P < 0.001 versus sham rats; ^#^ *P* < 0.05, ^##^ *P* < 0.01, ^###^ *P* < 0.001 versus nephrectomized vehicle-treated rats.

Renal protein levels of the pro-apoptotic Bcl-2 family member Bax, the apoptotic inducer cytochrome *c* and caspase-3, and also the anti-apoptotic protein survivin were examined. As shown in Figure 10(a), the Bax level was significantly augmented in nephrectomized control rats, while Hachimi-jio-gan and Bakumi-jio-gan-treated groups showed a significantly reduced level of Bax protein. Cytochrome *c* and caspase-3 protein expressions were also higher in vehicle-treated nephrectomized rats, but these protein expressions in the Bakumi-jio-gan-treated group were slightly or significantly decreased (Figures 10(b), 10(c)). In contrast, Hachimi-jio-gan-treated nephrectomized rats exhibited a significantly higher expression of survivin than vehicle-treated nephrectomized rats (Figure 10(d)).

### 3.10. Renal Histological Findings

**Figure 11:**
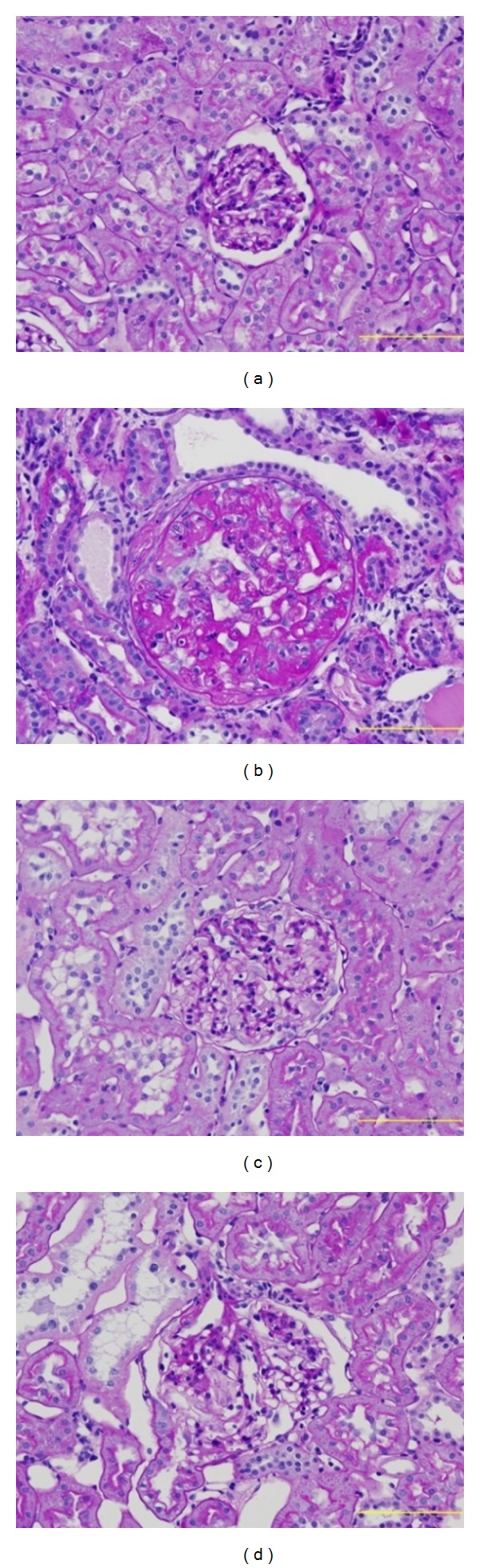
Photomicrographs of the glomeruli. (a) Sham rats, (b) nephrectomized vehicle-treated rats, (c) nephrectomized Hachimi-jio-gan-treated rats, and (d) nephrectomized Bakumi-jio-gan-treated rats. ×200.

Table 1 summarizes the results of the histological examination of the kidney. The severity of renal damage was evaluated by assigning lesion scores, as described in Materials and Methods. The renal tissue of the nephrectomized control rats indicated typical morphological changes in glomerular, tubular, and interstitial lesions due to the resection of 5/6 of their kidney volume. However, the indices of glomerular sclerosis were reduced in the Hachimi-jio-gan-treated groups compared with those in the sham group. While the glomerular sclerosis score of control rats was 3.19, the score of the rats given Hachimi-jio-gan was significantly reduced to 2.33. In addition, Hachimi-jio-gan and Bakumi-jio-gan prevented the tubulointerstitial damage. The score of control rats was 3.64, whereas those of rats given Hachimi-jio-gan and Bakumi-jio-gan were 1.72 and 2.66, respectively. Moreover, the rats given Hachimi-jio-gan and Bakumi-jio-gan had respective arteriolar sclerotic lesion scores of 1.24 and 1.65, while the nephrectomized control rats' score was 3.99. These results were reflected by the reduction of the total score, which was expressed as the sum of the scores of the three lesions, showing that Hachimi-jio-gan and Bakumi-jio-gan inhibited the development of these renal lesions. However, the ameliorated effect against the development of renal lesion was stronger in the Hachimi-jio-gan-treated groups. The typical glomerular morphology seen in the examined kidneys is illustrated in Figure 11.

## 4. Discussion

A reduction of the renal mass by subtotal nephrectomy in animals or by kidney disease in humans results in proteinuria, glomerulosclerosis, tubulointerstitial injury, and progressive deterioration of kidney function and structure. This process is mediated by a constellation of hemodynamic events, namely, glomerular hypertension and hyperfiltration, and non-hemodynamic events including oxidative stress, inflammation, and apoptosis. The prevailing oxidative stress in animals and humans with chronic renal insufficiency leads to the oxidation of proteins, carbohydrates, nucleic acids, lipids, and lipoproteins and accumulation of their harmful by-products in various tissues and body fluids [14–16]. In addition, the increased generation of ROS leads to tissue injury and dysfunction by attacking, denaturing, and modifying structural and functional molecules and by activating redox-sensitive transcription factors and the signal transduction pathway. These events, in turn, promote necrosis, apoptosis, inflammation, fibrosis, and other disorders. Oxidative stress and inflammation are constant features of advanced renal disease and play a major role in progressive deterioration of the renal function and structure and associated cardiovascular and numerous other complications of chronic kidney disease [17]. Therefore, we firstly investigated the effect of Hachimi-jio-gan and Bakumi-jio-gan on oxidative stress and ROS-related factors involved in the development of renal disease using nephrectomized rats.

 Although the origin of increased ROS generation in renal disease is multifactorial, studies have focused on the fact that NADPH oxidase mainly participates in the process of ROS generation [18]. There is accumulating evidence that non-phagocytic NADPH oxidases are major enzymatic sources of ROS generation in ischemia-reperfusion injury, inflammation, hypertension, and atherosclerosis based on experimental animal and human studies [19, 20]. Structurally, NADPH oxidase comprises a membrane-associated cytochrome *b*_558_, composed of one p22^phox^ (phox stands for phagocyte oxidase) and one gp91^phox^ subunit and at least four cytosolic subunits (p47^phox^, p67^phox^, p40^phox^, and the small GTPase rac1 or rac2) [21]. Especially, among them, Nox-4 and p22^phox^ were found to be a major source of ROS production in the tissue or cell, and could play a role under pathological conditions [22–24]. Therefore, we examined the protein expressions of Nox-4 and p22^phox^, subunits of NADPH oxidase, in the kidney to identify the exact mechanism in the prescription-treated group. Immunoblotting results showed that Nox-4 and p22^phox^ protein expressions were up-regulated in nephrectomized rats. In contrast, the Bakumi-jio-gan administration group showed a significant inhibition of these renal protein expressions, and the Hachimi-jio-gan administration group also showed down-regulated Nox-4 protein. However, the effects on subunits of these NADPH oxidase proteins were stronger in the Bakumi-jio-gan-treated group.

 On the other hand, oxidative stress induces alterations in the Nrf2 complex, and its gene transcription, such as that of HO-1, is enhanced [25]. Nrf2 is a redox-sensitive transcription factor that plays a vital role in protection against oxidant- and xenobiotic-induced cellular injury [26–28]. NADPH oxidase-derived ROS and the consequently induced activation of intracellular protein kinase cascades, such as mitogen-activated protein kinase, can mediate the induction of Nrf2 and HO-1 expressions [29, 30]. Therefore, the Nrf2-HO-1 pathway could be a biomarker of oxidative stress and an adaptive response under pathological conditions. In our results, nephrectomized rats showed decreased expressions of Nrf2 and HO-1 in the remnant kidney compared with sham rats; however, Bakumi-jio-gan administration effectively alleviates oxidative stress and results in the up-regulation of Nrf2 and HO-1.

 JNK is activated by a number of cellular stimuli including proinflammatory cytokines and ROS. Hojo et al. [31] demonstrated JNK activation by ROS in endothelial cells via the modulation of cellular protection systems against ROS. It is speculated that JNK plays an important role in signal events through the phosphorylation of c-Jun, activation of AP-1, and stimulation of proinflammatory gene expression such as ICAM-1. In addition, ROS is induced from JNK [31]. JNK is involved in the regulation of gene expression and stabilization through phosphorylation of JNK, p-JNK [32]. Only p-JNK, which is translocated to the nucleus, activates c-Jun/AP-1 [33]. TGF-*β*-mediated fibronectin induction requires the activation of JNK which, in turn, modulates the activity of c-Jun [34]. In this study, we demonstrated that JNK, p-JNK, c-Jun/AP-1, and TGF-*β* proteins were significantly increased in nephrectomized rats compared with sham rats. On the other hand, the administration of Hachimi-jio-gan markedly suppressed expressions of these proteins.

 NF-*κ*B is one of the cross-talk points of multiple signal transduction pathways, playing a key role in the regulation of transcription and expression of many genes such as COX-2 and iNOS, involved in inflammatory responses [35–37]. In the nephrectomized model, severe and progressive renal injury was associated with increased activity of NF-*κ*B, whereas amelioration of proteinuria and renal structural damage were associated with decreased activity of the NF-*κ*B system. Moreover, enhanced oxidative stress stimulates the expression of MCP-1 and ICAM-1. Macrophage infiltration regulated by adhesion molecules, MCP-1 and ICAM-1, is known to be associated with glomerular immune complex deposition and increased kidney chemokine production [38, 39]. The present study demonstrated that renal NF-*κ*Bp65, COX-2, iNOS, MCP-1, and ICAM-1 protein expressions were significantly enhanced in nephrectomized compared to sham rats. In contrast, Bakumi-jio-gan administration for 10 weeks significantly suppressed NF-*κ*Bp65 and ICAM-1 proteins, and augmented MCP-1 protein was slightly decreased by 150 mg/kg of Bakumi-jio-gan. The administration of Hachimi-jio-gan led to a significant down-regulation of COX-2 and iNOS expressions, whereas its prescription did not significantly alter the NF-*κ*Bp65, MCP-1, and ICAM-1 levels. Therefore, these findings suggest that NF-*κ*B and its related inflammatory molecules including proinflammatory cytokines, chemokines, and adhesion molecules may be critical factors involved in the effect of Bakumi-jio-gan.

 ROS, generated from NADPH oxidase and/or mitochondrial metabolism, exert a significant effect on various redox-sensitive signaling processes, for example, cell growth, apoptosis, migration, and extracellular matrix modeling [40], and contribute to apoptosis in podocytes and mesangial and tubular cells [41, 42]. Cell apoptosis induces cell death and, eventually, loss of function in tissues due to mitochondrial dysfunction including membrane potential loss, the up-regulation of Bax, and release of cytochrome *c* [43]. It has been shown that Bax may influence permeability and the release of cytochrome *c* from inter-membrane spaces into the cytosol, while survivin is able to stabilize and inhibit membrane pore opening, ultimately protecting against oxidative stress [44]. Cytochrome *c* release from mitochondria is a critical step in the apoptotic cascade and this activates downstream caspases such as caspase-3, which is implicated in the pathogenesis of renal injury and may be blocked by antioxidants [45, 46]. In this study, the administration of Hachimi-jio-gan and Bakumi-jio-gan in nephrectomized rats significantly suppressed renal protein expressions of Bax and caspase-3, although there were no significant changes in the cytochrome *c* protein level, indicating that these two prescriptions protected against renal mitochondrial dysfunction through the suppression of apoptotic proteins in nephrectomized rats. However, survivin expression in Hachimi-jio-gan-treated rats exhibited a significant up-regulation from the value of nephrectomized control rats. The results presented here suggest that Hachimi-jio-gan could have a crucial effect against apoptosis and anti-apoptosis in renal tissue in the presence of nephrectomy.

 Proteinuria over a long period due to subtotal nephrectomy causes irreversible structural changes in nephrons and impairment of renal function, that is, glomerular-capillary hypertension, proximal tubule damage, activation of nuclear signals such as NF-*κ*B, releasing vasoactive and inflammatory substances into the interstitium, interstitial inflammatory reaction, and consequent fibrosis [47, 48]. Moreover, increased glomerular function caused by renal ablation is triggered by glomerular hyperfiltration, and enlargement and destruction of the glomerulus, eventually leading to glomerular sclerosis [49]. This observation showed that nephrectomy induced the early stage of chronic renal failure with changes in the glomerulus, including increased mesangial cell proliferation, glomerular capillary endothelial damage and matrix production, as well as tubulointerstitial injury. In addition, chronic renal failure enhances a number of well-established risk factors for atherosclerosis (e.g., blood pressure, plasma levels of atherogenic lipoproteins) [50]. In our histological examination, too, clear histological lesions of the increased severity of glomerular, tubular, interstitial, and arteriolar lesions were observed under nephrectomy, whereas, in nephrectomized rats given Hachimi-jio-gan and Bakumi-jio-gan orally, the glomerular and tubulointerstitial damage was ameliorated. The arteriolar sclerotic lesion was also ameliorated. Although the ameliorating effect on the development of renal lesions was stronger in the Hachimi-jio-gan-treated group, the two prescriptions play a role in improving renal pathological lesions by nephrectomy.

 On the other hand, it is considered that uremic toxins retained under renal dysfunction directly or secondarily affect renal tissue, leading to a deterioration of renal tissue and function, producing a vicious cycle that results in the terminal stage of kidney disease [51]. In this connection, the present study shows that the serum urea nitrogen and Cr levels were increased significantly in nephrectomized rats; however, Hachimi-jio-gan and Bakumi-jio-gan-administered nephrectomized rats showed significantly lower levels. The removal of a toxin that affects the renal function might have a beneficial effect on maintaining the cellular function. However, it remains unclear whether this improvement is a primary effect, or is secondary to or occurs simultaneously with amelioration of the histological improvements.

 According to traditional Chinese medicine theory, chronic renal failure is generally considered to be Qi-Yang deficiency, that is, energy exhaustion, accompanied by Xie-Qi repletion. The clinical treatment principle is to replenish Qi-Yang and drain off Xie-Qi [52]. The two prescriptions used in this experiment included both Chinese medicinal principles: complementary herbs and eradicating herbs. In addition, Hachimi-jio-gan and Bakumi-jio-gan are composed of Rokumi-jio-gan, one of the most effective agents for complementary prescription. To compare the effectiveness of these two prescriptions, we focused on oxidative stress, inflammation, and apoptosis, which was advanced in rats that had undergone excision of five-sixths of their kidney volume. The administration of Hachimi-jio-gan to subtotal nephrectomized rats exerted a favorable influence on the prevention of renal failure progression, at least in part, through amelioration of the JNK pathway and apoptosis-induced renal damage, whereas Bakumi-jio-gan exerted a protective effect against the amelioration of renal oxidative stress- and inflammation-related protein.

 Although the detailed mechanisms of Hachimi-jio-gan and Bakumi-jio-gan were not clarified in the present study, our findings may support therapeutic evidence for the two prescriptions ameliorating the development of renal failure. In addition, further study of the biological role of individual component drugs and main components has to be supported, even though it is cautiously proposed that the effect of Hachimi-jio-gan or Bakumi-jio-gan against renal failure would probably result from the synergistic effect of the crude drugs as well as the effect of component drugs.

## 5. Conclusions

Two Rokumi-jio-gan-containing prescriptions (Hachimi-jio-gan and Bakumi-jio-gan) played a protective role in the progression of renal failure. The administration of Hachimi-jio-gan to subtotal nephrectomized rats exerted a favorable influence on the prevention of renal failure progression, at least in part, through amelioration of the JNK pathway and apoptosis-induced renal damage, whereas Bakumi-jio-gan exerted a protective effect against the amelioration of renal oxidative stress- and inflammation-related protein.

## Figures and Tables

**Table 1:** Histopathological evaluation of the kidney.

Group	Glomerular sclerosis (Score)	Tubulointerstitial damage (Score)	Arteriolar sclerotic lesion (Score)	Total (Score)
Sham	0.00 ± 0.00	0.00 ± 0.00	0.00 ± 0.00	0.00 ± 0.00
Nx-Veh	3.19 ± 0.16**	3.64 ± 0.16**	3.99 ± 0.04**	12.38 ± 0.08**
Nx-HJG	2.33 ± 0.08^∗∗, #^	1.72 ± 0.16^∗∗, ###^	1.24 ± 0.18^∗, ###^	5.27 ± 0.10^∗, ###^
Nx-BJG	2.60 ± 0.05**	2.66 ± 0.06^∗∗, ##^	1.65 ± 0.03^∗∗, ###^	7.20 ± 0.05^∗∗, ###^

Sham, sham rats; Nx-Veh, nephrectomized vehicle-treated rats; Nx-HJG, nephrectomized Hachimi-jio-gan-treated rats; Nx-BJG, nephrectomized Bakumi-jio-gan-treated rats. Data are the mean ± S.E.M. Significance: *P < 0.01, **P < 0.001 versus sham rats; ^#^ *P* < 0.05, ^##^ *P* < 0.01, ^###^ *P* < 0.001 versus nephrectomized vehicle-treated rats.
